# Speaker Adaptation on Articulation and Acoustics for Articulation-to-Speech Synthesis

**DOI:** 10.3390/s22166056

**Published:** 2022-08-13

**Authors:** Beiming Cao, Alan Wisler, Jun Wang

**Affiliations:** 1 Department of Electrical and Computer Engineering, University of Texas at Austin, Austin, TX 78712, USA; 2 Department of Speech, Language, and Hearing Sciences, University of Texas at Austin, Austin, TX 78712, USA; 3 Department of Mathematics and Statistics, Utah State University, Logan, UT 84322, USA; 4 Department of Neurology, Dell Medical School, University of Texas at Austin, Austin, TX 78712, USA

**Keywords:** articulation-to-speech synthesis, silent speech interface, speaker adaption, voice conversion

## Abstract

Silent speech interfaces (SSIs) convert non-audio bio-signals, such as articulatory movement, to speech. This technology has the potential to recover the speech ability of individuals who have lost their voice but can still articulate (e.g., laryngectomees). Articulation-to-speech (ATS) synthesis is an algorithm design of SSI that has the advantages of easy-implementation and low-latency, and therefore is becoming more popular. Current ATS studies focus on speaker-dependent (SD) models to avoid large variations of articulatory patterns and acoustic features across speakers. However, these designs are limited by the small data size from individual speakers. Speaker adaptation designs that include multiple speakers’ data have the potential to address the issue of limited data size from single speakers; however, few prior studies have investigated their performance in ATS. In this paper, we investigated speaker adaptation on both the input articulation and the output acoustic signals (with or without direct inclusion of data from test speakers) using the publicly available electromagnetic articulatory (EMA) dataset. We used Procrustes matching and voice conversion for articulation and voice adaptation, respectively. The performance of the ATS models was measured objectively by the mel-cepstral distortions (MCDs). The synthetic speech samples were generated and are provided in the supplementary material. The results demonstrated the improvement brought by both Procrustes matching and voice conversion on speaker-independent ATS. With the direct inclusion of target speaker data in the training process, the speaker-adaptive ATS achieved a comparable performance to speaker-dependent ATS. To our knowledge, this is the first study that has demonstrated that speaker-adaptive ATS can achieve a non-statistically different performance to speaker-dependent ATS.

## 1. Introduction

Laryngectomees are people who have their larynx partially or totally removed in surgeries (laryngectomy), due to the treatment of laryngeal cancer [1]. Especially, for people who have undergone total laryngectomy, their ability to produce normally voiced speech is lost. Currently, they have three main options for their daily communication: esophageal speech [2], tracheo-esophageal puncture (TEP) speech [3] and electro-larynx (EL) [4,5]. The major common disadvantage of these approaches is they generate unnatural or hoarse voices, which discourages their use and causes social isolation [6]. Silent speech interfaces (SSIs) are devices that enable speech communication when a human’s phonatory abilities are impeded [7,8,9]. SSIs convert (silent) articulatory motion to speech, which have the potential of recovering speech ability for people who are unable to produce speech sounds but are still able to articulate(e.g., laryngectomees). There are currently two algorithmic designs in silent speech interfaces: the recognition-and-synthesis and the directly articulation-to-speech (ATS) synthesis. The recognition-and-synthesis design [7,10] recognizes textual information from non-audio articulatory signals with silent speech recognition [11], and then use a text-to-speech to convert recognized text to speech [12]. ATS is a procedure that directly maps a human’s articulatory bio-signals to speech. As an end-to-end model and compared to the recognition-and-synthesis design, ATS has become a popular software design for silent speech interfaces, because of its advantages of low-latency and easier implementation [8].

Currently, most of the ATS studies focus on speaker-dependent (SD) design, in which only the data from testing speakers are used to train the ATS model [13]. SD-ATS usually suffer the restriction from insufficient training data since it is difficult to record a large amount of articulatory data from the same speakers. The main reason is the current articulatory information capture approaches normally require directly [14,15,16] or indirectly [17,18] attaching hardware such as sensors to subjects’ articulators. Hours of data recording sessions will generally cause subjects to fatigue. Compared to speaker-dependent systems, speaker-independent (SI) systems require no training data from testing speakers [13] by using data collected from other speakers for training. Speaker-independent systems could be a solution for insufficient training data from individual subjects. However, due to the inter-speaker variability, they usually suffer lower performance than well-trained SD systems. Therefore, speaker adaption approaches may be an alternative solution for ATS. Speaker adaptation approaches adapt speaker-independent systems to the target speakers (users) [13], which take the advantages of both speaker-independent (large training dataset) and speaker-dependent (target speaker information involved) systems. Speaker adaptation approaches have been actively studied and demonstrated to be effective in automatic speech recognition (ASR) and text-to-speech (TTS) applications [13,19], but have been relatively less studied in ATS [20,21]. To highlight these concepts and assist the description in this paper, we list the major difference between these terminologies below.

- Speaker-dependent ATS (SD-ATS) is where training and testing data are from the same speakers;
- Speaker-independent ATS (SI-ATS) is where training and testing data are from different speakers;
- Speaker-adaptive ATS (SA-ATS) is where training data are from other speakers and the target speaker.

Speaker adaptation for ATS is challenging because the inter-speaker variations take place in both the input articulation and the output acoustics. In addition, to maintain the identity of the output speech from SSI, the output side of ATS (speech voice) has to be as similar as possible to the target speaker’s original voice. This characteristic restricted the usage of some averaging-based [19] and warping-based speaker adaptation approaches on the output audio, such as cepstral mean and variance normalization (CMVN) [22] and vocal tract length normalization (VTLN) [23]. A valid approach to perform adaptation on acoustic output is setting adapting other speakers’ acoustic data to the target speaker [24]. Therefore, in this study, we proposed a voice conversion-based audio adaptation approach for ATS.

In this study, we performed an investigation on speaker adaptation of ATS with voice conversion [24] and Procrustes matching [11]. The dataset used was a publicly available, electromagnetic articulograph (EMA) and audio data set (Haskins Production Rate Comparison database) [25]. The experiments were conducted in three sessions. The first session is the speaker-independent ATS (SI-ATS) as the baseline performance, and the speaker-dependent ATS as the target performance. Then we applied speaker adaptation on acoustics and articulation to the SI-ATS. In this session, Procrustes matching [11,26,27] was applied for the adaptation of the articulation, and voice conversion [24] models were adopted to convert the acoustic features of the training speakers to that are similar to the target speakers. Finally, we directly added the both articulatory and acoustic data of the target speakers to the training set to train a kind of speaker-adaptive (SA) model, then applied voice conversion and Procrustes matching on that to see if it could further improve the performance.

The ATS and voice conversion models used are long short-term memory (LSTM)-recurrent neural network (RNN). The Waveglow vocoder [28] was employed as the vocoder to convert the predicted acoustic features to speech waveforms. Due to the real-time decoding preference of ATS, advanced sequence-to-sequence models were not used in this study. Audible speech samples were generated and presented from the best ATS models in each experiment stage. Detailed discussions were made based on the experimental results.

The contributions of this paper include: (1) proposed and verified applying voice conversion for acoustics adaptation for speaker-independent (SI) ATS; (2) validated the Procrustes matching in SI-ATS application, which has only been shown effective in speaker-independent silent speech recognition [11,29]; (3) applied Waveglow vocoder [28] in EMA-based ATS application for the first time; (4) presented audible synthetic speech samples that were generated from multi-speaker (speaker-independent and speaker-adaptive) ATS.

## 2. Related Works

In the silent speech interface area, multiple techniques have been used for capturing articulatory motion data for the SSI purpose including: electromagnetic articulograph (EMA) [10,11,14,15], permanent magnet articulograph (PMA) [16,30,31,32], ultrasound image (UI) [18,20,33,34], surface electromyography (sEMG) [17,35], non-audible murmur (NAM) [36]. Doppler signals have been explored in the SSI application as well [37,38]. Kapur et al. used neuromuscular signals captured with electrodes as the input of SSI [39]. Recently, frequency-modulated continuous-wave radar has been investigated for SSI application as well [40]. Sebkhi et al. [41] have proposed an inertial measurement unit (IMU)-based PMA device that is suitable for SSI usage. As mentioned previously, most of the studies above used a speaker-dependent design, one of which is speaker-dependent and session-independent [35].

Only a few recent works studied speaker-independent and speaker-adaptive ATS systems. Shandiz et al. [20] have conducted studies on embedding speaker information into the ultrasound-based ATS to improve the performance on multiple speakers, in which the data from the testing speakers were involved in the training set. Similarly, Ribeiro et al. [21] also conducted multi-speaker ATS with ultrasound image data for a validation of their newly proposed dataset. The authors of [42] presented a study on speaker-independent mel-cepstrum estimator, in which the speaker-independent acoustic feature estimator was improved by embedding d-vectors and using pre-averaged acoustic. This study focused on speaker-independent systems [42], but the model predicted mel-cepstrum coefficients only, without generating speech samples. Although these ATS performances have been improved, no one has achieved a comparable performance by speaker-dependent ATS. In addition, no previous study was able to generate audible speech samples in their SI- or SA-ATS models.

This present study explored speaker-independent ATS and generated speech samples from that. The speaker adaptation was performed in a strategy of adapting voice from training speakers to that from the targeting speakers, which requires training one specific ATS model for one target speaker. This strategy is different to that in [20,42], which embedded speaker information to train one ATS model that aims to work for all testing speakers.

## 3. Dataset

The dataset used in this study is a dataset collected by the Haskins Lab, Yale University [25], which is an open access dataset, in which the electromagnetic articulography (EMA) data [14] and audio data were synchronously recorded from eight native American English speakers (four males, four females). The stimuli are the 720 phonetically balanced Harvard sentences from [43]. Each speaker read the 720 sentences at least two times, one in a normal speaking rate, one in a fast speaking rate. After that, they read a varying number of sentences in the normal speaking rate. In total, 1553 to 1738 sentences were recorded from each speaker, the duration of recorded data from each speaker is about 1 h. Additional details on the amount of data available for each speaker are provided in Table 1.

The EMA data were recorded with the NDI Wave system, 8 sensors were attached to the tongue tip (TT), tongue blade (TB), tongue rear (TR), upper lip (UL), lower lip (LL), mouth left (corner) (ML), jaw, and jaw left (canine) [25]. Three-dimensional (x: posterior –> anterior, y: right –> left, z: inferior –> superior) articulatory movement of the sensors were recorded in a sampling rate of 100 Hz. The trajectories of sensors have been filtered with a 20 Hz Butterworth lowpass filter after recording. The audio data were recorded at a sampling rate of 44,100 Hz. In this study, we used 6 of 8 sensors for the experiments: tongue tip (TT), tongue blade (TB), tongue rear (TR), upper lip (UL), lower lip (LL), and jaw (JAW), which is consist with the setup in the mngu0 EMA dataset [44]. The audio data were downsampled from 44,100 Hz to 22,050 Hz, to make it consistent with the trained Waveglow vocoder [28] used in this study.

Other than the dataset used in this study, EMA-MAE corpus [45] is another EMA dataset that was collected from multiple speakers. EMA-MAE corpus is the EMA dataset that was collected from a relatively large number of speakers (40 speakers in total). About 30 to 45 min data were collected from each speaker, and part of that are isolated words. Therefore, the EMA-MAE dataset was not used in this study, due to the smaller amount of data from single speakers.

## 4. Methods

### 4.1. Articulation-to-Speech Synthesis

**Figure 1:**
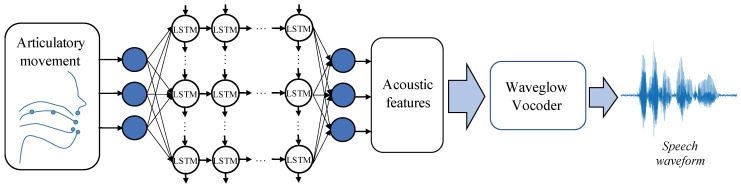
The overview illustration of a generic articulation-to-speech synthesis model.

Figure 1 provides an overview of the implementation of articulation-to-speech synthesis models in this study. Articulatory movement of articulators (tongue, lips and jaw) was captured with sensors and sampled into frames, then fed to the ATS to predict the acoustic feature for speech synthesis. To maintain the real-time implementation of ATS, the advanced sequence-to-sequence models were excluded in this study. The ATS model used in this study is the long short-term memory-recurrent neural networks (LSTM-RNN), which has been shown to outperform typical deep neural networks (DNN) [15,42]. The bidirectional-LSTM (BLSTM) model has high performance in preliminary experiments, but the BLSTM-based ATS models do not support real-time SSI implementation.

The vocoder used in this study is the Waveglow vocoder, which is a flow-based network capable of generating high-quality speech from mel-spectrograms [28]. WaveGlow combines insights from the invertible implementation Glow [46] and the high performance neural vocoder WaveNet [47]. It has been demonstrated that WaveNet could generate higher-quality speech samples than the conventional source-filter vocoders [12,47,48,49] but in relatively high latency. WaveGlow showed a similar performance to WaveNet, but in a very low latency [28]. In addition, [34] demonstrated that Waveglow vocoder outperformed conventional vocoders [50,51,52] in ultrasound image-based ATS. Therefore, WaveGlow vocoder was chosen as the vocoder in this study, and the trained Waveglow model for English (WaveGlow-EN) provided by NVIDIA was directly adopted without additional training.

The acoustic features are same as the default setup of Waveglow which were 80-dimensional mel-spectrograms, the fast Fourier transform (FFT) size was 1024, hop size (step size) was 256. The articulatory data were consisted of the 3-dimensional (3D) spatial location of six sensors at a sampling rate of 100 Hz, as mentioned the sensors were attached to six articulators: tongue tip (TT), tongue blade (TB), tongue rear (TR), upper lip (UL), lower lip (LL), and jaw (JAW). The first- and second-order derivatives were concatenated to the movement frames as the input frames, therefore the dimension of the ATS input is 54 (3-dim. × 6 sensors × 3). Although the left–right dimension is not as significant as the other two dimensions (front-back, and up-down) in speech production, 3D EMA data have demonstrated higher performance the 2D in preliminary experiments. Finally, the articulatory data of each phrase were scaled to the same length to the extracted acoustic features accordingly by interpolation.

The experimental results were measured with the mel-cepstral distortions (MCDs) [53]. For the MCD computation, the mel-spectrogram features were converted to the mel-frequency cepstral coefficients (MFCC) by applying discrete cosine transform (DCT). With the first 13 MFCCs, the MCDs were computed with the Equation (1) [54], the first MFCC was not included in the computation since it represents system energy gain rather than speech quality information (Equation (1)). In Equation (1), $C_{m,d}$ indicates the d-th (1≤d≤D) MFCC dimension at time step *m* (0≤m≤T). *D* is equal to 13, which is the total dimensional of MFCC included. *T* is the total number of MFCC frames generated.

(1) $$\begin{array}{c} MCD=\frac{10}{ln10}\sum {{}_{m=0}}^{T}\sqrt{2\sum_{d=1}^{D}{\left(C_{m,d}-C_{m,d}^{gen}\right)}^{2}} \end{array}$$

### 4.2. Acoustic Adaptation Using Voice Conversion

**Figure 2:**
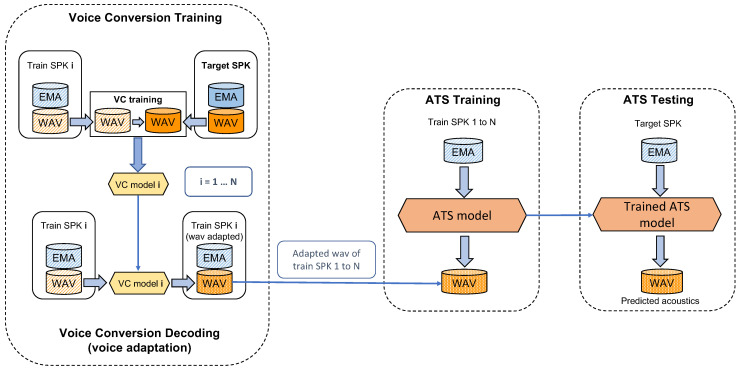
The pipeline of ATS Speaker adaptation using voice conversion. For each target speaker, the other N (seven) speakers were training speakers.

Voice conversion (VC) is a type of voice transformation which aims to convert speech utterances of a source speaker to sound as if it was uttered by a target speaker [55]. Therefore, VC could be a suitable technology for adapting the voice of training speakers to the target speakers’ voice [24]. Figure 2 shows the schema of the VC-based speaker adaptation for a single target speaker. The eight speakers take turns to be the target speaker in the cross-validation loop. Then train voice conversion models with the phrases in the training set of target and training speakers. The acoustic features of parallel phrases were aligned to the same length by the dynamic time warping (DTW) [56]. With the aligned acoustic features, VC models were trained for each of the target-training speaker pairs. After that, the acoustic features of training speakers were converted to target speakers’ acoustic features by the VC models, and used for the speaker-independent ATS model training.

### 4.3. Articulation Adaptation Using Procrustes Matching

**Figure 3:**
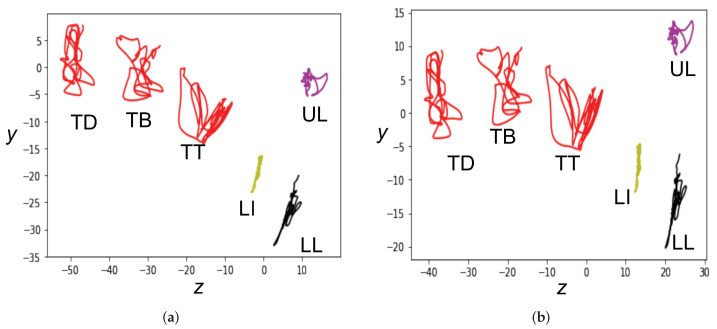
Example of shapes (motion path of the articulators) before and after Procrustes matching for producing “the birch canoe slid on the smooth planks”. In this coordinate system, *y* is vertical and *z* is anterior-posterior. (**a**) Before Procrustes matching. (**b**) After Procrustes matching.

Procrustes matching [27] is a robust statistical two-dimensional shape analysis technique [29,57]. In Procrustes analysis, shapes are composed of ordered series of landmarks on articulators (Figure 3a,b). Shapes from different participants have different sizes, relative locations, and different angles of tongue and lips, which leads to inter-speaker variations. In this study, Procrustes matching was conducted in *y* (vertical) and *z* (anterior-posterior) dimensions, which reduced the inter-speaker physiological difference. Procrustes matching has shown improvement in the silent speech recognition studies [11,29,57]. In this study, we applied Procrustes matching to all the EMA data as a normalization method for ATS. Specifically, for instance, let $\left(y_{i},z_{i}\right)$ represents the *i*-th data point (spatial coordinates) of a sensor, then for each sentence the speaker spoke, the data points will construct a set of landmarks *S* (sensors). *S* can be represented as below:

(2) $$S=\{\left(y_{i},z_{i}\right)\},\;i=1,\ldots ,n$$

*n* is the total number of data points. As mentioned, *y* is the vertical direction and *z* is the front-back direction. A full procedure of Procrustes matching includes: (1) translating all articulatory data of each speaker to the average position of all data points in the shape (averaged across speaker); (2) rotating all shapes of each speaker to the angle that the centroids of lower and upper lips movements defined the vertical axis [57]; (3) scaling all shapes to unit size. Previous tests indicated that scaling will cause a slight increase in the error rate in silent speech recognition, therefore scaling was eliminated from the Procrustes matching approach in this experiment. The translation and rotation operations in Procrustes matching are described with the equation below:

(3) $$\left[\begin{array}{c} \bar{y_{i}}\\ \bar{z_{i}} \end{array}\right]=\left[\begin{array}{cc} \cos\theta & -\sin\theta \\ \sin\theta & \cos\theta \end{array}\right]\left[\begin{array}{c} \beta_{y}\\ \beta_{z} \end{array}\right]\left[\begin{array}{c} y_{i}-c_{y}\\ z_{i}-c_{z} \end{array}\right]$$

$\left(c_{y},c_{z}\right)$ are centroids of the two shapes which were used as translation factors; $\left(\beta_{y},\beta_{z}\right)$ are the square roots of the sum of the squares of all data points along the *y* and *z* directions; θ is the angle to rotate [27]. An example of Procrustes matching is provided in Figure 3. Figure 3a illustrates the original motion trajectories of a sample speaker when producing the phrase “the birch canoe slid on the smooth planks”. Figure 3b illustrates those same trajectories after Procrustes matching has been used to align them to those of a separate speaker.

Procrustes matching could be applied at two levels: sentence-level and speaker-level. Sentence-level is to obtain the parameters in Equation (2) from the same sentences produced by different speakers respectively. The speaker-level obtains the parameters from all sentences produced by one speaker. During testing for both levels, individual (test) shapes were translated and rotated according to the obtained parameters. Preliminary results have shown that sentence-level Procrustes matching outperforms the speaker-level matching. Therefore, only sentence-level Procrustes matching was reported in this paper.

## 5. Experimental Setup

In the ATS experiments of this study, 50 sentences from each speaker’s data were used as the testing set, another 50 sentences as the validation set, and the rest for training. The eight speakers took turns being chosen as the target speaker, and the other seven speakers were used as training speakers (leave-one-subject-out cross-validation). As introduced, the experiments in this study were conducted in three sessions: (1) speaker-independent (SI) and speaker-dependent (SD) ATS; (2) speaker adaptation for speaker-independent ATS on the acoustic (output) and articulation (input); (3) speaker-adaptive (SA) experiments by adding target speakers’ data to the training set with and without further applying the speaker adaptations in session (2). In the speaker-dependent experiment, the model was trained, validated and tested with the same speakers. The speaker-independent experiments trained and validated models with seven training speakers, then tested with the left eighth speaker. The speaker-adaptive experiments directly adding the data from testing speakers to the training set of SI, validated and tested with data from testing speakers. The validation here indicates hyper-parameter exploration with the validation sets.

The detailed experimental setup of the deep learning models in this study were presented in Table 2. As mentioned, we use LSTM-RNN for the ATS model to maintain the real-time function of SSI, and BLSTM-RNN for the VC models for speaker adaptation. The training of all models was conducted in a batch size of single whole sentences. ATS models take 54-dim. EMA data as input and predict 80-dim. mel-spectrograms for Waveglow vocoder. To achieve the best baseline performance of both SD- and SI-ATS models before our improvement approaches (VC and Procrustes matching), we used distinct hyper-parameters for them, including learning rates and max epochs. The hyperparameters were chosen in a preliminary experiment, where a grid search of two to six layers LSTM and 128 to 512 nodes was performed. The hyper-parameter setups with the best performance were selected. Both input and output of VC were 80-dim. mel-spectrogram. All deep learning models were implemented with the Pytorch toolkit [58].

### 5.1. Speaker-Dependent (Target) and Speaker-Independent (Baseline) ATS

We firstly conducted speaker-dependent (SD) and speaker-independent (SI) ATS experiments for all speakers as the target (ceiling) and baseline performances, respectively. The speaker-dependent ATS uses training, validation, and testing data from the same speakers. Although no inter-speaker variation in SD experiments, normalization on the input articulatory data could help accelerate training and improve performance. Therefore, SD-ATS with and without z-score normalization were performed. The z-score normalization on the input EMA data was conducted by firstly computing the dimension-wise mean and standard deviation (STD) from the training set, then applying the mean and STD to the training, validation, and testing set (*X*_norm_ = (X− mean)/STD). Preliminary results have indicated that z-score normalization provides consistent improvement on the SD-ATS performance.

In speaker-independent ATS experiments, the training data are the mixture of training sets from seven training speakers. To maintain the concept of speaker-independent, the validation data are the 50-sentence validation set of training speakers (7 speakers × 50 = 350 sentences). Same as SD-ATS, z-score normalization improved SI-ATS as well. As mentioned, we also applied Procrustes matching on the input EMA data. One thing that is worth noting is that when applying both of them together (z-score and Procrustes matching), the translation operation in Procrustes matching was eliminated by the z-score normalization, thus only the rotation operation affected the performance. In addition, z-score normalization will be applied in all following experiments by default since it has been demonstrated effective.

### 5.2. Acoustic Adaptation for SI-ATS Using Voice Conversion

Starting from the baselines speaker-independent ATS (with and without Procrustes matching), we adopted voice conversion models for acoustic adaptation (Figure 2). For the purpose of developing high performance and easy implementation, we used parallel voice conversion models in which the data from the source and target speaker shared the same stimulus. Across all eight speakers, 1428 parallel phrases were found in the dataset. These 1428 phrases were used for the VC model development, in which we use 14 for validating VC model training, 14 for testing, and the rest 1400 for training.

The eight speakers took turns to be the target speaker in the cross-validation loop. Figure 2 shows the pipeline of the VC-based speaker adaptation for a single target speaker. Firstly we trained voice conversion models with the phrases in the training set of target and training speakers. The acoustic features of parallel phrases were aligned to the same length by the dynamic time warping (DTW) [56]. With the aligned acoustic features, VC models were trained for each of the target-training speaker pairs. The VC models were bi-directional LSTM (Table 2) since no real-time implementation was required at this stage (voice conversion), and the BLSTM outperformed LSTM in the preliminary experiment. After that, the acoustic features of training speakers were converted to target speakers’ acoustic features by the VC models, and used for the later multi-speaker ATS model training.

The speaker-independent ATS experiments with this VC speaker adaptation were essentially not speaker-independent, since the audio data from the target speakers were used during the adaptation (VC). However, for the convenience for describing the different setups and for distinguishing the ATS experiments in which both articulation and audio data from the target speaker were used for training, we still call these experiments “speaker-independent with voice conversion” in the rest of this paper (SI-VC in Results section).

### 5.3. Speaker Adaptive ATS including Training Data from Target Speakers

In this session, we directly added the training set from target speakers to the dataset that trained speaker-independent with and without voice conversion, for a further speaker adaptation to see if that could outperform speaker-dependent ATS (target performance). As mentioned we named this a type of speaker-adaptive model in this study (SA). The Procrustes matching (after z-score normalization) was used by default in this stage. In this session, we maintained the method with target speakers, in which one ATS model was trained for one target speaker (rather than one ATS model that works for all speakers). The main difference was the validation sets were from the current target speaker, rather than all of them. After that, we applied the voice conversion approach on this SA-ATS.

## 6. Results

**Figure 4:**
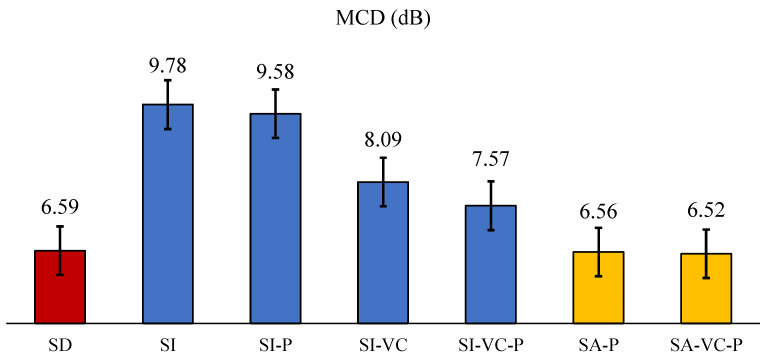
Average MCDs of the experiments in this study. **SD**: speaker-dependent. **SI**: speaker-independent. **SI-P** speaker-independent with Procrustes matching. **SI-VC**: speaker-independent ATS with voice conversion. **SA**: data from targets speakers were directly added to the ATS training set.

Figure 4 shows the average mel-cepstral distortions (MCDs) across all speaker and Table 3 details the MCD values of each speaker. Note that lower MCD values generally indicate that the speech output of the ATS model is more similar to the participant’s actual speech, and thus indicates a higher performance. As can be observed, on average, the speaker-independent ATS with Procrustes matching (SI-P) outperforms that without Procrustes matching (SI), across all speakers except M01 and M03. Speaker-independent ATS with voice conversion adaptation (SI-VC) showed consistent improvement in the speaker-independent experiments (Figure 4). When both of the Procrustes matching and voice conversion were applied, we saw additional improvements in MCD (SI-VC and SI-VC-P). After adding the testing speakers’ data to the ATS training set (SA-P), the average MCD decreased significantly and slightly outperformed speaker-dependent ATS (on average). Voice conversion brought further improvement (SA-VC-P), but much less dramatic than in speaker-independent experiments. Procrustes matching was used here by default since it was verified effective for speaker normalization in the previous session. A Mann-Whitney U test indicated the significant difference of the proposal SA approaches (SA-P and SA-VC-P) outperformed the baseline approach (SI) (*p* < 0.001 for both SA-P and SA-VC-P) and there were no significant differences with the target performance (SD).

**Figure 5:**
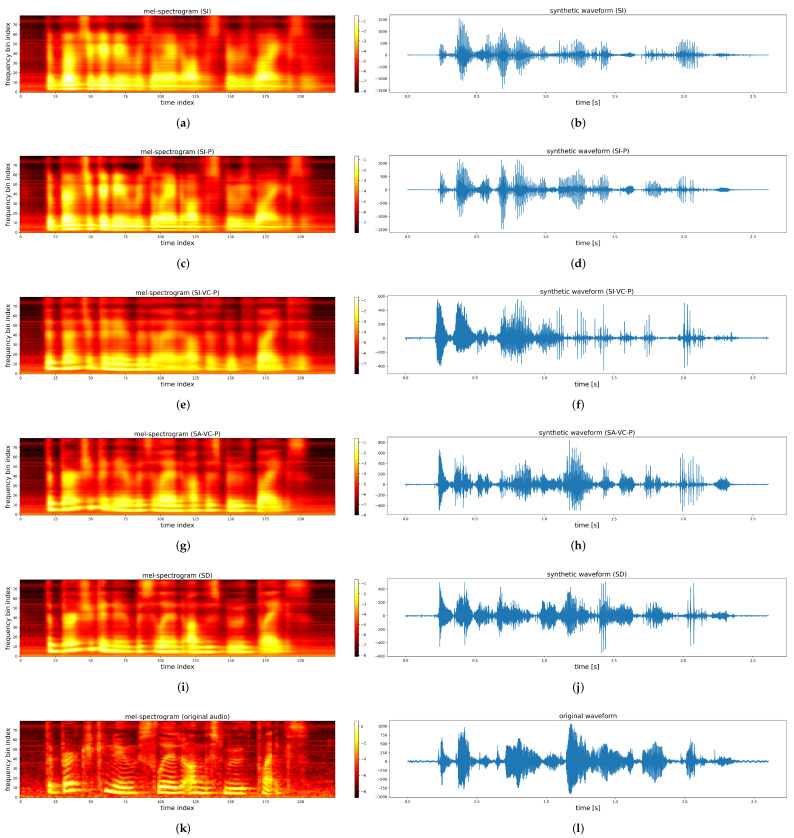
Examples of original and speaker-dependent ATS predicted mel-spectrograms and the synthetic waveforms. (**a**) Mel-spectrogram from SI ATS. (**b**) Speech waveform from SI ATS. (**c**) Mel-spectrogram from SI-P ATS. (**d**) Speech waveform from SI-P ATS. (**e**) Mel-spectrogram from SI-VC-P ATS. (**f**) Speech waveform from SI-VC-P ATS. (**g**) Mel-spectrogram from SA-VC-P ATS. (**h**) Speech waveform from SA-VC-P ATS. (**i**) Mel-spectrogram from SD ATS. (**j**) Speech waveform from SD ATS. (**k**) Original mel-spectrogram. (**l**) Original speech waveform.

The audio speech samples were generated from the experiments [59]. Figure 5 provides illustrations of the predicted (or original) mel-spectrogram and the synthetic speech waveforms from different ATS models including speaker-independent ATS (with and without Procrustes matching), ATS with speaker adaptation of voice conversion (with and without directly adding the training set from target speakers), and the speaker-dependent ATS. Visually, it appears that the most significant improvements in the frequency resolution were brought by the voice conversion and directly adding the testing speakers’ data to the training set since there is less visible stratification across the harmonics in the SI-ATS and SI-P-ATS spectrograms. By contrast, the difference in frequency resolution and synthetic waveform between SI and SI-P, SA-VC-P, and SD are not equivalently significant. Selected synthetic speech samples are available at [59]. Speech samples from a speaker-independent (SI) ATS have rarely been presented. Perceptually, the SI-ATS speech samples in this study sound like audible but less intelligible speech.

The MCDs of voice conversion across all source-target speaker pairs were presented in Table 4. The values in this table tell us the similarities between the source speakers and the target speakers following the voice conversion process (lower MCD ≈ more similar). Speakers with more similar speech characteristics will likely exhibit lower MCD values.

## 7. Discussion

### 7.1. Acoustic and Articulation Adaptation Performances

Voice conversion has brought more significant improvement than the Procrustes matching (Table 3). The Procrustes matching has brought additional and consistent improvement when combined with voice conversion. Speaker-independent ATS has speaker variation in both the input articulation and output acoustics. Therefore, it is natural that adapting both articulation and acoustics outperform adapting only one of them. The Procrustes matching is an average-based normalization approach, while the voice conversion in this study is a “personalized” adaptation that converts all training speakers’ voice to that of the target speakers. Therefore, it is expected that voice conversion improved speaker-independent (SI) ATS more than the Procrustes matching. In practice, the voice conversion approach proposed in this study is expected to reduce the effort of articulatory data collection, since it only adopts audio data from the target speakers. Collecting acoustic data only is less challenging than collecting synchronized acoustic and articulatory data. Audio data could also be collected remotely, which is normally impractical for current SSI articulatory data collection approaches. In addition, voice conversion requires less training data than ATS, audible speech could be generated by a VC model trained with only 10–20 sentences [24].

As shown in the results, the inclusion of the data (both acoustic and articulatory) from target speakers is a dominating advantage in training ATS (SA approaches), which has significantly outperformed the speaker-independent experiments. The VC adaptation has also shown less improvement here (6.56 –> 6.52 dB). Although not statistically significant, both SA-P and SA-VC-P outperformed speaker-dependent ATS on average. It is worth noting that the performance of each approach still varied significantly across speakers, as seen in Table 3. Although the performance differences were somewhat marginal, they illustrate the potential efficacy of speaker adaptation methods. These results demonstrated that the data (both acoustic and articulatory) from target speakers is still a strong advantage in training ATS, which also indicated the challenge in outperforming SD-ATS with speaker adaptation approaches. Further improvements in the effectiveness of both SI and SA methods are likely to come as datasets with larger groups of speakers. Such as in speaker-independent ASR systems that generally use tens or hundreds of speakers in their training data.

It notes that, although our speaker-adaptive ATS obtained comparable performances with SD-ATS, it does not mean speaker-adaptive ATS could outperform SD-ATS with an increased number of subjects and data size from single speakers. As the number of subjects increases, the inter-speaker variability in both articulation and acoustics increases. Although SD-ATS may be still the first choice when developing new ATS algorithms, our findings suggest SA-ATS may be a promising alternative solution.

### 7.2. Performance Variation across Speakers

Given the similar data amount, the eight speakers in the dataset have shown different performances in both SD- and SI-ATS (Table 3). Due to the inter- and intra-speaker variation, the MCDs have shown obvious differences across speakers in all experiments. Speakers with lower intra-speaker variation may show higher performance in speaker-dependent ATS. Speakers that have higher similarity to other speakers seem to have higher performance in speaker-independent ATS (e.g., F01). Speakers’ data with higher intra- and inter-speaker variation may demonstrate lower performance in SD and SI experiments, respectively (e.g., M01 and M02).

### 7.3. Observations from the Synthetic Speech Samples

Interestingly, in the synthetic speech samples presented in [59], it was observed that the speaker-independent ATS generated “gender-confused” speech samples. We expect this because the training set includes data from both genders. While the speech samples from speaker-independent ATS with VC adaptation show obvious gender characteristics since the training data from the opposite gender were converted. Therefore, the voice conversion adaptation may also improve the gender characteristic in the synthetic speech. Gender-dependency in speaker-independent ATS might be a topic that is worth further investigation in the future.

### 7.4. Relationship between VC and ATS Performances

Table 4 has shown the MCDs of voice conversion model development. The rows and columns are the source and target speakers of voice conversion. The Pearson correlation coefficients between the mean VC performance (Table 4) and the SI-VC performance across all speakers (Table 3) was 0.84 and was decreased to 0.78 for the correlation between VC and SI-VC-P (Table 3). The improvement from Procrustes matching reduced the correlation between VC and the SI-VC-P. It is possible that an SI-ATS model for a target speaker got strong acoustics adaptation by the voice conversion, while the Procrustes matching for that speaker was not strong enough accordingly to form a good mapping between the adapted articulation and the acoustics. Therefore, a matched adaptation of acoustics and articulation might be more effective in the task of speaker adaptation for ATS.

### 7.5. Feasibility of Articulation Conversion

Other than the Procrustes matching, an alternative approach for articulation adaptation is the articulation conversion, which has a similar procedure to the voice conversion in this study. In the articulation conversion, the articulatory data from the training speakers were converted to that of target speakers with the trained articulation conversion models. However, the articulation conversion generated articulatory movement with a spatial RMSE larger than 3 mm, which led to a performance decrease in the following ATS experiments. The EMA data are low-frequency time domain signals, which might be more challenging to precisely predict than the high-frequency frequency domain acoustic features. As an end-to-end model, ATS might be very sensitive to the variation in the input articulation. Therefore, we did not use articulation conversion in the current study. More studies are needed to confirm the feasibility of this articulation conversion approach.

## 8. Conclusions

In this study, we investigated speaker adaptation approaches for articulation-to-speech synthesis using voice conversion and Procrustes matching. Procrustes matching was first applied to reduce the speaker variations in the articulation. Then a framework of using voice conversion for ATS voice adaptation was proposed and validated, in which voice conversion (VC) models were trained for reducing the acoustic variations between training and testing speakers. The experimental results have shown the effectiveness of both Procrustes matching and voice conversion; the performance was further improved when both were used in conjunction. Additionally, we performed speaker-adaptive (SA) ATS experiments in which the data from the target speakers (both acoustic and articulatory) were included in the training set (both with and without VC adaptation) and achieved a similar performance to the speaker-dependent ATS. To our knowledge, this is the first study that demonstrated the potential of speaker-adaptive ATS by showing a comparable performance to that of speaker-dependent ATS. This study is also the first to demonstrate audible speech output from speaker-independent and speaker-adaptive ATS systems.

## Figures and Tables

**Table 1:** Number of sentences and duration recorded from each speaker.

Speaker	Phrase Num.	Duration (min)
F01	1738	61.75
F02	1560	60.58
F03	1617	58.63
F04	1618	59.71
M01	1553	55.67
M02	1554	57.47
M03	1610	60.31
M04	1620	59.65
Sum.	12,870	472.82
Ave.	1609	59.22

**Table 2:** Topologies of the neural networks in this study.

**Acoustic Feature**	
Mel-spectrogram	80-dim. vectors
Sampling rate	22,050 Hz
Windows length	1024
Step size	256
**Articulatory Feature**	54-dim. vectors
Articulatory movement (6 sensors)	(18-dim. vectors) + Δ + ΔΔ (54-dim.)
**SD-ATS LSTM Topology**	
Input	54-dim. articulatory
Output.	80-dim. acoustic feature
No. of LSTM nodes each hidden layer	256
Depth	3-depth layers
Batch size	1 sentence (one whole sentence per batch)
Max Epochs	50
Learning rate	0.0003
Optimizer	Adam
**SI-ATS LSTM Topology**	
Input	54-dim. articulatory
Output.	80-dim. acoustic feature
No. of LSTM nodes each hidden layer	256
Depth	3-depth layers
Batch size	1 sentence (one whole sentence per batch)
Max Epochs	30
Learning rate	0.00001
Optimizer	Adam
**VC BLSTM Topology**	
Input	80-dim. acoustic feature
Output.	80-dim. acoustic feature
No. of LSTM nodes each hidden layer	128
Depth	3-depth layers
Batch size	1 sentence (one whole sentence per batch)
Max Epochs	30
Learning rate	0.00005
Optimizer	Adam
**Toolkit**	Pytorch

**Table 3:** MCD of ATS experiments on each speaker.

	SD	SI	SI-P	SI-VC	SI-VC-P	SA-P	SA-VC-P
**Train:**	**Tar SPK**	**Src SPK**	**Src SPK (P)**	**VC-Src SPK**	**VC-Src SPK (P)**	**Src + Tar SPK (P)**	**Tar + VC-Src SPK (P)**
**Test:**	**Tar SPK**	**Tar SPK**	**Tar SPK (P)**	**Tar SPK**	**Tar SPK (P)**	**Tar SPK (P)**	**Tar SPK (P)**
F01	4.98	7.80	7.48	6.63	5.79	5.26	5.08
F02	5.47	8.41	8.21	6.82	6.45	5.51	5.23
F03	6.02	9.04	8.66	8.03	6.99	6.11	6.20
F04	5.99	8.37	8.35	7.87	7.19	6.14	6.33
M01	8.96	10.41	10.44	9.45	9.33	8.22	8.23
M02	7.54	10.66	10.05	9.25	8.85	7.29	7.21
M03	6.59	8.18	8.37	7.95	7.55	6.87	6.85
M04	7.14	8.83	8.69	8.71	8.38	7.11	7.03
Mean	6.59	8.96	8.78	8.09	7.57	6.56	6.52
STD	1.27	1.04	0.98	1.03	1.21	0.99	1.05

**Table 4:** MCD of voice conversion (dB) during the speaker adaptation on acoustics. The diagonal cells are empty, because voice conversion is not applicable to the same speaker.

	Target	F01	F02	F03	F04	M01	M02	M03	M04
Source									
F01			6.32	7.23	6.71	7.08	6.86	7.91	8.69
F02		9.26		6.75	7.60	7.66	7.55	8.46	9.15
F03		7.43	7.01		7.02	6.85	7.04	7.83	8.77
F04		7.15	6.24	7.45		7.24	7.66	8.02	9.25
M01		6.64	6.40	6.32	7.38		7.03	7.68	8.52
M02		6.47	6.64	6.50	7.56	6.78		7.77	8.70
M03		6.97	6.63	6.65	7.51	6.70	7.05		8.50
M04		7.01	6.32	7.05	7.42	7.96	7.17	7.71	
Average		7.30	6.51	6.85	7.31	7.18	7.19	7.91	8.80

## Data Availability

Publicly available datasets were analyzed in this study. This data can be found here: https://yale.app.box.com/s/cfn8hj2puveo65fq54rp1ml2mk7moj3h/folder/30415804819 (accessed on 30 June 2022).
